# Inert Coats of Magnetic Nanoparticles Prevent Formation of Occlusive Intravascular Co-aggregates With Neutrophil Extracellular Traps

**DOI:** 10.3389/fimmu.2018.02266

**Published:** 2018-10-02

**Authors:** Rostyslav Bilyy, Harald Unterweger, Bianca Weigel, Tetiana Dumych, Solomiya Paryzhak, Volodymyr Vovk, Ziyu Liao, Christoph Alexiou, Martin Herrmann, Christina Janko

**Affiliations:** ^1^Danylo Halytsky Lviv National Medical University, Lviv, Ukraine; ^2^Section of Experimental Oncology and Nanomedicine (SEON), Department of Otorhinolaryngology, Head and Neck Surgery, Else Kröner-Fresenius-Stiftung Professorship, Universitätsklinikum Erlangen, Erlangen, Germany; ^3^Master Programme in Advanced Materials and Processes, Technische Fakultät, Friedrich-Alexander-Universität Erlangen-Nürnberg, Erlangen, Germany; ^4^Department of Internal Medicine 3 - Rheumatology and Immunology, Friedrich-Alexander-Universität Erlangen-Nürnberg and Universitätsklinikum Erlangen, Erlangen, Germany

**Keywords:** neutrophil extracellular traps (NETs), superparamagnetic iron oxide nanoparticles (SPIONs), biocompatibility, vascular occlusion, clearance, nanoparticle aggregation

## Abstract

If foreign particles enter the human body, the immune system offers several mechanisms of response. Neutrophils forming the first line of the immune defense either remove pathogens by phagocytosis, inactivate them by degranulation or release of reactive oxygen species or immobilize them by the release of chromatin decorated with the granular proteins from cytoplasm as neutrophil extracellular traps (NETs). Besides viable microbes like fungi, bacteria or viruses, also several sterile inorganic particles including nanoparticles reportedly activate NET formation. The physicochemical nanoparticle characteristics fostering NET formation are still elusive. Here we show that agglomerations of non-stabilized superparamagnetic iron oxide nanoparticles (SPIONs) induce NET formation by isolated human neutrophils, in whole blood experiments under static and dynamic conditions as well as *in vivo*. Stabilization of nanoparticles with biocompatible layers of either human serum albumin or dextran reduced agglomeration and NET formation by neutrophils. Importantly, this passivation of the SPIONs prevented vascular occlusions *in vivo* even when magnetically accumulated. We conclude that higher order structures formed during nanoparticle agglomeration primarily trigger NET formation and the formation of SPION-aggregated NET-co-aggregates, whereas colloid-disperse nanoparticles behave inert and are alternatively cleared by phagocytosis.

## Introduction

Nanoparticles have attracted increasing attention for biomedical applications. Especially superparamagnetic iron oxide nanoparticles (SPIONs) can be used as contrast agent in magnetic resonance imaging (MRI), as drug transporter for magnetic drug targeting (MDT) or as magnetizer for cells in magnetic tissue engineering. To ensure a safe use in biomedicine, the interaction of SPIONs with components of the human blood system must be warranted. For host defense and biocompatibility, neutrophils play a major role. They are the most frequent leukocyte type in human blood, representing more than 65% of all white blood cells. In inflammatory conditions, neutrophils are the first type of leukocyte that migrates toward the site of insult where they produce inflammatory mediators and chemoattractants. To eliminate foreign pathogens, neutrophils have a broad range of antimicrobial functions (1). For instance, activated neutrophils can phagocytose, release antimicrobial granules, and produce reactive oxygen species (ROS) during a process referred to as oxidative burst. Neutrophils catch and immobilize pathogens by the release of neutrophil extracellular traps (NETs), composed of extracellular decondensed DNA, covered with nuclear histones and granular antimicrobial proteins, preventing the spread of pathogens and initiating their inactivation (2).

The neutrophils' defense mechanisms reportedly occur not only for viable pathogens, but also for sterile nanoparticles (3). Interestingly, amongst others, size dependent effects decide by which mechanism particles are cleared from the body. For nanodiamonds it has been shown that very small nanoparticles (10–40 nm) induce fast damage of plasma membranes and instability of the lysosomal compartment, leading to the immediate formation of NETs, whereas larger particles (100–1,000 nm) behaved rather inertly (3). Fungal hyphae and yeasts are cleared via NETosis and phagocytosis, respectively (4).

For SPIONs, data on the size dependency of the neutrophil response are lacking. This is true for *in vitro* experiments as well as for *in vivo* experiments mimicking the intended clinical use. Thus, for application of SPIONs as drug transporters for magnetic drug targeting, the interactions of SPIONs have to be analyzed in the presence of a magnetic field. Here we show that in the absence of an appropriate coating nanoparticles tend to form irreversible agglomerates, prone to cause NET formation, vascular occlusion and thrombotic events. Coating the SPIONs with dextran or albumin prevented agglomeration, NET formation and vascular occlusions. We conclude that coating of SPIONs is required for safe biomedical applications, especially if the particles are applied intravascularly.

## Materials and methods

### Synthesis of superparamagnetic iron oxide nanoparticles (SPIONs) and coating

Lauric acid-coated iron oxide nanoparticles were synthesized using a co-precipitation method as described by Tietze et al. (5). In brief, Fe(II) and Fe(III) salts were dissolved in water, then NH_3_ solution 25% was added under stirring. SPIONs were coated with lauric acid (LA) *in situ* or afterwards after washing, respectively.

For coating with LA afterwards, the precipitate was washed with 1.3% ammonium hydroxide solution, and then LA (dissolved in acetone) was added and the whole dispersion was heated to 90°C for 4 min under stirring. The resulting LA-coated SPIONs (SPION^LA1^) were washed 10 times with 1.3% ammonium hydroxide solution.

For *in situ* coating with LA, after precipitation of the particles by NH_3_ and heating to 90°C, 1.25 g LA solution (dissolved in acetone) was added, cooled down and dialyzed (SPION^LA2^) (6). SPION^LA2^ were further stabilized with human serum albumin according to Zaloga et al. (SPION^LA−HSA^) (6). Briefly, AlbIX solution (10% w/V, Albumedix, Nottingham, England) was dialyzed (MWCO 8 kDa, Spectra/Por® 6) against 4.5 l of ultrapure water (4 water changes, 5 h). Tangential ultrafiltration (MWCO 30 kDa) was used to concentrate the solution to the original volume. Subsequently, 10 ml of the respective albumin solution were stirred with 200 rpm at room temperature and SPION^LA2^ was added dropwise through a 0.8 μm syringe filter to receive a total iron concentration of 2.5 mg/ml. After 10 min stirring, excess albumin was removed by tangential ultrafiltration (7).

Dextran-coated SPIONs (SPION^DEX^) were synthesized according to Unterweger et al. with slight modifications (8). In brief, FeCl_3_ and FeCl_2_ (molar ratio Fe^3+^ /Fe^2+^ = 2) were added to an aqueous solution containing 8.8% (w/w) dextran. Addition of ammonia to the ice cold solution led to the precipitation of the particles. The suspension was heated to 75°C for 45 min and afterwards cooled to room temperature. Particles were purified by dialysis and ultrafiltration. The dextran shell was cross-linked with epichlorohydrin under basic conditions to increase particle stability. Finally, particles were purified by dialysis and ultrafiltration. All nanoparticle solutions were sterile filtered using syringe filters and the total iron content was determined employing microwave plasma atomic emission spectroscopy. Nanoparticles were previously characterized physicochemically; basic features are summarized in Table 1.

**Table 1:** Physicochemical characterization of SPIONs.

**SPION name**	**Stock conc. (mg/ml)**	**Size by dynamic light scattering (DLS) (nm)**	**Zeta potential (mV)**
SPION^LA1^	4.41	127.9	−30.7
SPION^LA2^	11.96	55.8	−25.1
SPION^LA−HSA^	5.16	58.9	−11.9
SPION^DEX^	5.74	31.0	−1.7

### Preparation of human material

All analyses of human material were performed in full agreement with institutional guidelines and with the approval of the Ethical Committee of the University Hospital Erlangen (permission number 257_14B). Platelet-rich plasma was generated by centrifuging lithium-heparin anticoagulated venous whole blood from normal healthy volunteers at 200 g for 10 min. Polymorphonuclear cells (PMN) were obtained by density gradient centrifugation using Lymphflot (Bio-Rad Medical Diagnostics GmbH, Dreieich, Germany) as described elsewhere (9). In brief, whole blood was diluted with phosphate buffered saline (PBS) and pipetted carefully onto Lymphflot solution and centrifuged for 20 min at 850 g without brake (acceleration 1, deceleration 0). Then, the plasma and peripheral blood mononuclear cells (PBMC) layer were discarded. The PMN-rich layer on top of the erythrocytes was collected and erythrocytes were removed by hypotonic lysis. Cell viability and cell count were determined by MUSE cell analyzer (Merck-Millipore, Billerica, MA, USA).

### Animal experiments

Studies involving animals, including housing and care, euthanasia, and experimental protocols were conducted in accordance with the local animal ethical committee in the animal house of Danylo Halytsky Lviv National Medical University, permission number 5/23.02/17, under the supervision of a certified veterinary doctor. Ten white laboratory rabbits, 4-month old males were used for this investigation. Rabbits were housed in individual cages in a temperature/humidity/light-controlled environment, with both food and drinking water available *ad libitum*. Specifically, a rabbit was injected with sedative, afterwards fixed and 500 μg of SPION nanoparticles were injected into the central artery of the ear. Injection site was 2–3 cm from the base of the ear. Central artery goes in the middle of the era, then providing left and right marginal veins. A neodymium magnet (5 cm in diameter) was placed just below one of the marginal veins (more external), while the other vein was gently pressed at the base of the air, to limit the blood flow and direct all the blood flow to the vein with the placed magnet. The ear was immobilized on the magnet for at least 20 min after SPION injection; then the vessels were examined for clottings. If needed, the animal was sacrificed and ear tissues were fixed in 4% paraformaldehyde (PFA), then embedded and processed for hematoxylin and eosin (HE) staining and immune histochemistry using previously described techniques (10).

### Sterility and endotoxin content of SPIONs

For sterility testing, nanoparticles were diluted in H_2_O to receive concentrations of 250, 50, and 10 μg/ml. H_2_O and saliva (diluted 1/5 and 1/25) served as negative and positive controls, respectively. 100 μl of test sample were plated onto the agar plates in duplicates, and incubated at 37°C for 72 h. After 72 h, petri dishes were analyzed for growing colonies and documented by photography. Endotoxin-content of the nanoparticles was analyzed by EndoZyme endotoxin test (Hyglos, Bernried, Germany) according to the manufacturer‘s instructions. Nanoparticles were tested in concentrations of 25 and 50 μg/ml in endotoxin-free water. Endotoxin spiked samples served as controls. To 100 μl of standards and samples, reaction mixture (consisting of enzyme, substrate, assay buffer) was added and the reaction was monitored for 90 min at 37°C in a Microplate Reader Filter Max F5 (Molecular Devices; Biberach an der Riss, Germany; excitation 360 nm/emission 465 nm). The endotoxin amount [EU/ml] of the samples was calculated according to the standard curve parameters. Assay was accepted when spiking recovery of the samples was between 50 and 200%.

### Incubation of SPIONs with PMN or whole blood

Nanoparticles were taken up in plasma or PBS in a concentration of 400 μg/ml and pre-incubated for 10 min. PMN were taken up in PBS or plasma in a density of 4 × 10^6^/ml. Fifty microliters nanoparticles and 50 μl PMN were pipetted together in small FACS tubes, resulting in a final nanoparticle concentration of 200 μg/ml and a PMN density of 2 × 10^6^/ml and incubated for 3 h at 37°. Analogous, PMN were taken up in RPMI medium containing 10% FBS and incubated with nanoparticles. NET formation was stimulated with 100 ng/ml Phorbol myristate acetate (PMA, Sigma Aldrich, St Louis, MO, USA). After incubation, cells were fixed with 100 μl 1% PFA in PBS.

One hundred microliters whole blood were incubated with 200 μg/ml nanoparticles for 3 h at 37°C in small FACS tubes. After 3 h, erythrocytes were lysed by formic acid (pH 2.7) and pH was reconstituted by a solution containing sodium carbonate, sodium chloride and sodium sulfate (pH 11.2). Cells were washed with PBS, centrifuged and supernatant was discarded. The pellet was taken up in 100 μl 1% PFA in PBS. Cells were analyzed in flow cytometry or fluorescence microscopy.

### Incubation of PMN or whole blood with SPIONs in presence of magnet

Seven hundred and fifty microliters PMN (1 × 10^6^/ml) or 500 μl whole blood were filled into Eppendorf tubes. Magnets (diameter 0.5 cm) were glued to the vials using adhesive tape. Forty micrograms per milliliters SPIONs were added and shaken in horizontal position for 3 h at 37°C. NET formation was stimulated with 100 ng/ml PMA. After 3 h, the magnets were removed, the tubes were inverted several times and nanoparticle agglomerates were harvested. Smears from PMN were prepared on glass slides, stained with Sytox Green Nucleic Acid Stain (Therma Fisher Scientific, Waltham, MA, USA) washed, embedded with mounting medium and analyzed in fluorescence microscopy. Whole blood agglomerates were filtered with 70 μm cell strainers, washed repeatedly with PBS and embedded in Tissue Tek at −20°C for preparation of cryosections.

### Flow cytometry

PMN integrity was analyzed in flow cytometry using a Gallios cytofluorometer™ (Beckman Coulter, Fullerton, CA, USA). Viable PMN were identified and gated by forward scatter (FSC) and side scatter (SSC) properties. Data were analyzed employing Kaluza™ software Version 1.2 (Beckman Coulter, Fullerton, CA, USA) and processed in Microsoft Excel.

### Fluorescence microscopy

Cells were transferred to 96 well plates and centrifuged at 500 g for 5 min to sediment cells and NETs. Then, NETs were visualized by a Zeiss AxioObserver.Z1 fluorescence microscope (Carl Zeiss AG, Oberkochen, Germany). Pictures were processed with ZEN pro 2012 software (Carl Zeiss AG) and Adobe Photoshop.

### Immune histochemistry

Ten micrometers cryosections (embedded in Tissue Tek) were prepared and stored at −20°C until use. Then, sections were fixed with aceton-methanol (1:1) for 90 s. Cryosections were dried at room temperature for 5 min and blocked with 10% FBS in PBS for 1 h at room temperature. Cells on slides were permeabilized with 0.1% Triton X-100 in H_2_O for 10 min at room temperature. Primary antibody for neutrophil elastase (NE) (Abcam, United Kingdom, ab21595) 1:200 or for citrullated histone H3 (citH3) (Abcam, ab5103) 1:200 were added in 10% FBS in PBS overnight at 4°C. Slides were washed three times with PBS and incubated for 1.5 h at room temperature in the dark with secondary anti-rabbit IgG antibody conjugated with Cy5 (Jackson ImmunoReseach, Suffolk, United Kingdom, 11-175-144) 1:400 in H_2_O. Slides were washed with PBS three times and incubated with Sytox Green (2.5 μM) for 15 min at room temperature. Slides were washed with H_2_O three times and slides were embedded with DAKO fluorescent mounting medium (Dako, Hamburg, Germany). Samples without primary antibodies served as controls. Slides were analyzed in fluorescence microscopy.

## Results

Nanoparticles for application into the blood stream must fulfill several requirements. They have to be free of microbial or endotoxin contaminations, have to be stable as colloids and must not cause adverse (immune) reactions in the presence of blood cells and plasma components. Neutrophils are the most abundant cells in human blood and form the first line immune defense against invaders, like microbial, fungal or nanoparticular origin. In this study we investigated if SPIONs cause activation of neutrophils with concomitant NET formation in the presence and absence of magnetic fields. For that, SPIONs with different coatings, hydrodynamic sizes and physicochemical properties were applied, as summarized in Table 1. All used SPION systems were free of bacterial contaminations and endotoxin content was below 0.5 EU/mg (data not shown).

### *In vitro* formation of SPION-aggregated NET-co-aggregates in plasma, PBS, or medium containing 10% serum

**Figure 1:**
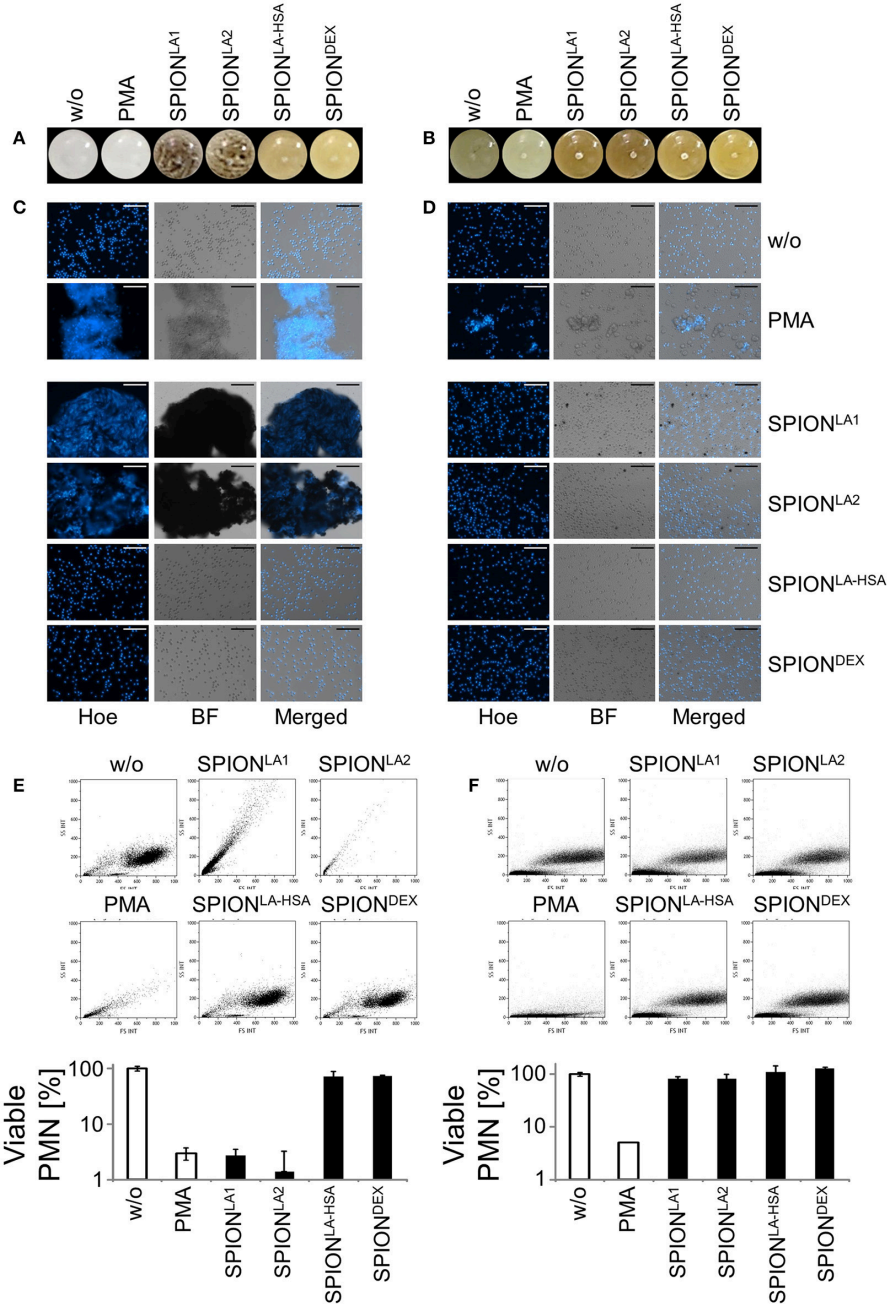
Non-stabilized SPIONs induce NET formation in protein-free buffer but not in plasma. In PBS non-stabilized SPIONs (200 μg/ml) form agglomerates **(A)**, whereas in plasma the particles are stabilized by protein coronae **(B)**. PMN were incubated with 200 μg/ml nanoparticles for 3 h in PBS **(C)** or plasma **(D)**. Then, samples were stained with Hoechst, and prepared for fluorescence microscopy. PMA treated and untreated cells served as positive and negative control, respectively. Scale bars refer to 100 μm **(C,D)**. Measurement of neutrophils by flow cytometry and evaluation of viable PMN count based on forward and side scatter properties in PBS **(E)** or plasma **(F)**. Experiment was performed in triplicates of at least two independent donors; representative data of one donor (mean values with standard deviations) are shown **(E,F)**.

**Figure 2:**
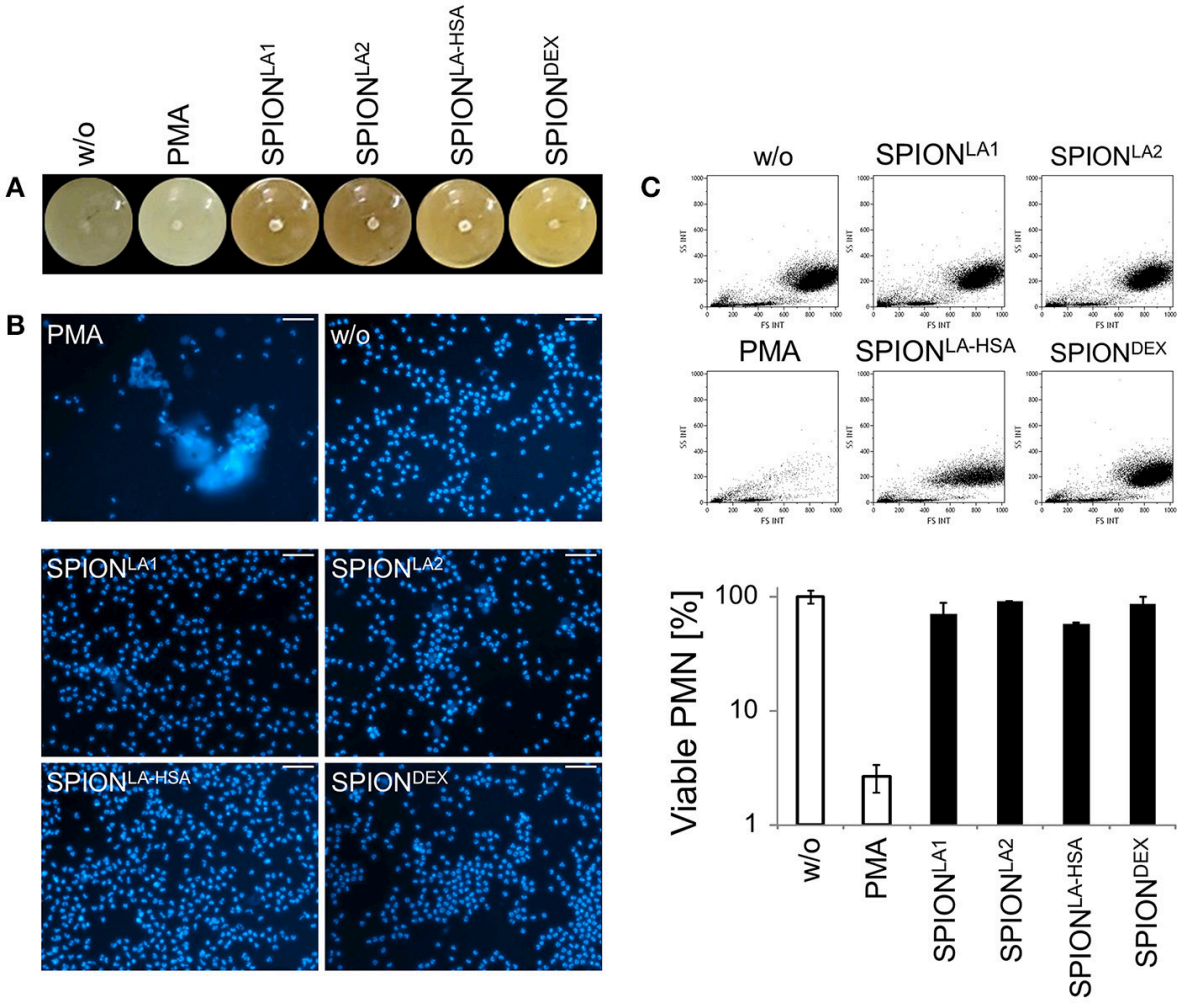
Serum reduced NET formation of isolated PMN. In serum-containing medium (R10) all SPIONs (200 μg/ml) are colloidally stable **(A)**. Isolated PMN were incubated with 200 μg/ml nanoparticles for 3 h in R10. Then, cells were stained with Hoechst, and prepared for fluorescence microscopy. PMA treated and untreated cells served as positive and negative control, respectively. Scale bars refer to 50 μm **(B)**. Measurement of neutrophils by flow cytometry and evaluation of viable PMN count based on forward and side scatter properties. Experiment was performed in triplicates of at least two independent donors; representative data of one donor (mean values with standard deviations) are shown **(C)**.

Two hundred micrograms per milliliters SPIONs were suspended in plasma, PBS or medium containing 10% serum and incubated for 10 min. We observed that the SPIONs exhibited different colloidal stabilities, with SPION^LA1^ and SPION^LA2^ forming clusters after incubation in the presence of PBS (Figure 1A); incubation with plasma or medium containing 10% serum did not induce clusters due to the formation of stabilizing protein coronae (Figures 1B, 2A) (11, 12).

After 3 h of incubation with PMN in PBS we found extracellular DNA in those samples in which nanoparticle aggregates had been observed (Figure 1C). PMA as canonical stimulator for NET formation also induced extracellular DNA in the presence of PBS. In plasma or serum-containing medium no NET formation was observed for SPIONs; NETs were induced by PMA, however, they were considerably smaller (Figures 1D, 2B). So far, it seems that formation of NETs in the presence of SPIONs depends either on the size of the nanoparticle agglomerates or on the medium (PBS, plasma or serum-containing medium). Analysing PMN with viable morphology by means of forward and side scatter we detected aggregated NETs (aggNETs) for SPION^LA1^ and SPION^LA2^ in PBS, but not for the other nanoparticles (Figure 1E). In the presence of plasma or medium containing 10% serum, no aggNETs were detected after incubation with SPIONs (Figures 1F, 2C).

### *In vitro* formation of SPION-aggregated NET-co-aggregates in media containing 10% serum in the presence of a magnetic field

**Figure 3:**
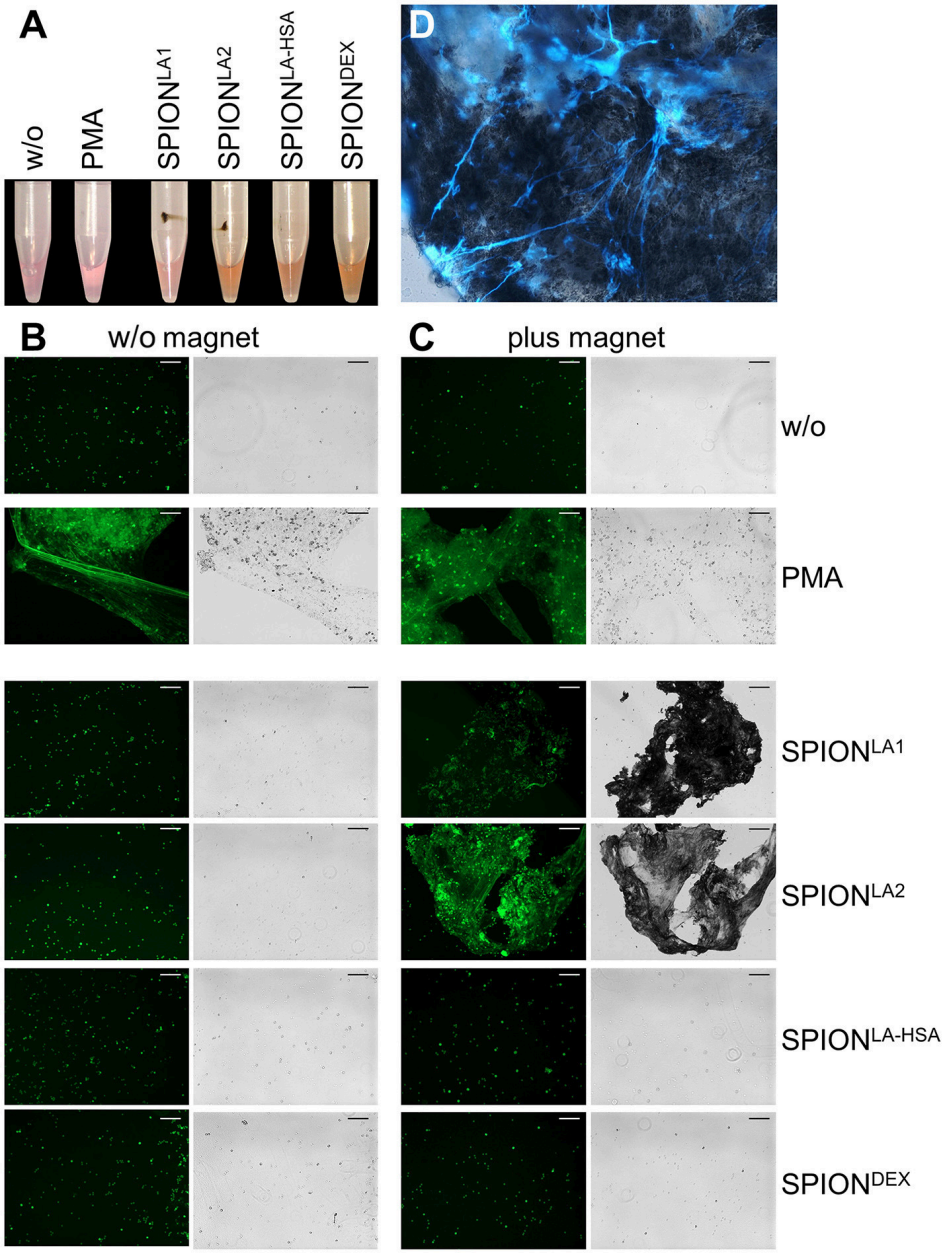
Magnetic fields increase NET formation of isolated PMN in serum-containing media. Isolated PMN were incubated with 40 μg/ml SPIONs in R10 media under constant shaking at 37°C in the absence or presence of magnetic fields. After 3 h, magnets were removed and the tube walls rinsed with medium **(A)**. The harvested cells were stained with Sytox Green and prepared for fluorescence microcopy. Scale bars refer to 100 μm **(B,C)**. Smear of the SPION^LA1^- induced aggNET-co-aggregate was stained with Hoechst 33342 **(D)**.

To analyze *in vitro* NET formation in media containing 10% serum PMN were incubated with 40 μg/ml SPIONs on an orbital shaker to prevent passive sedimentation of the nanoparticles. Then the samples were subjected or not to magnetic fields for 3 h under constant shaking at 37°C. In these conditions SPION^LA1^, SPION^LA2^, and SPION^LA−HSA^ accumulated close to the magnets. The SPION^LA−HSA^ conglomerates were instable and could easily be resuspended by shaking; those of SPION^LA1^ and SPION^LA2^ formed tight and stable aggregates that could not be resuspended by shaking. In the absence of magnets no agglomerations were detected (Figure 3A).

Centrifuged samples were stained for NETs. Fluorescence microscopy revealed fibers of extracellular/extranuclear DNA for SPION^LA1^ and SPION^LA2^ incubated in the presence of a magnetic field. These looked similar to NETs induced by PMA (Figure 3C), LPS, MSU crystals, and zymosan (not shown); in the absence of a magnetic field no extracellular DNA fibers were detected (Figure 3B). Brightfield microscopy showed dark nanoparticles entrapped in the NETs (Figure 3C). Fluorescence microscopy of a SPION^LA1^–induced aggNET-co-aggregate revealed DNA fibers around the nanoparticle agglomerate (Figure 3D).

### *Ex vivo* formation of SPION-aggregated NET-co-aggregates in whole blood in the presence of a magnetic field

**Figure 4:**
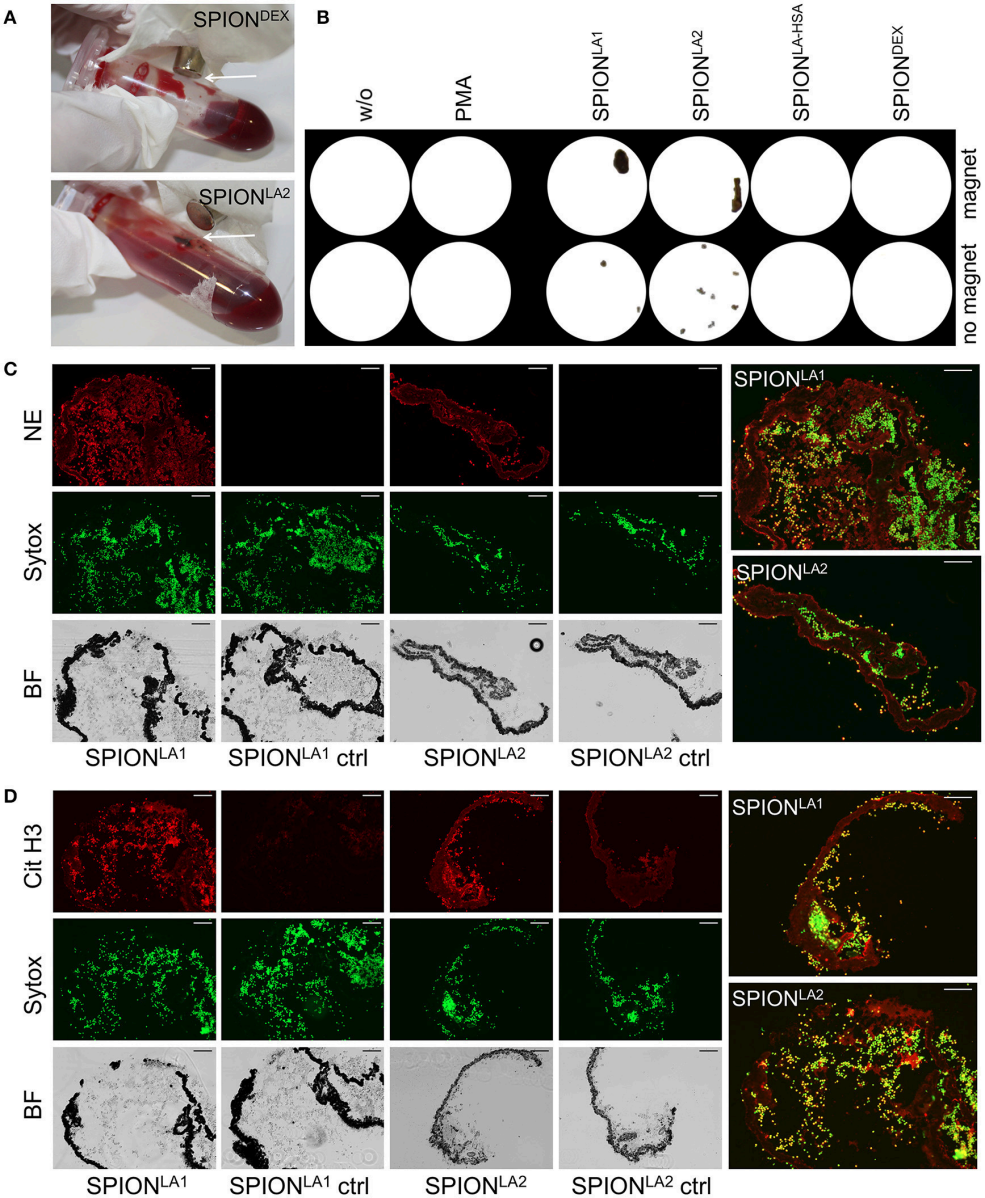
NETosis in whole blood in absence/presence of magnet. Whole blood was incubated with 40 μg/ml SPIONs under constant shaking at 37°C in the absence or presence of magnetic fields **(A)**. After 3 h, agglomerates were harvested on 70 μm mesh cell strainers and prepared for sectioning **(B)**. Cryosections (10 μm) of agglomerates were stained for neutrophil elastase (NE) **(C)** or citrinulated histone H3 (cit H3) **(D)** and analyzed in fluorescence microscopy. Scale bars refer to 100 μm. Large images represent overlays of the green and red fluorescences for SPION^LA1^ and SPION^LA2^ **(C,D)**.

Next we incubated whole blood with 40 μg/ml SPIONs in the absence or presence of a magnetic field. The samples were incubated on an orbital shaker. After 3 h we observed brown agglomerates sticking to the tube walls close to the magnets for SPION^LA1^ and SPION^LA2^, which were easily visible by the naked eye. For SPION^DEX^ and SPION^LA−HSA^ no visible agglomerates were detected (Figure 4A). To analyze the agglomerates in more detail, we isolated the large structures employing a 70 μM mesh (Figure 4B). In the absence of a magnetic field, no large agglomerates were formed; we only detected small brown structures for SPION^LA1^ and SPION^LA2^. PMA induced NETs appeared whitish since they did not contain dark brown nanoparticles. Cryosections of the agglomerates were stained for neutrophil elastase (NE), citrullinated histone H3 (citH3), and extracellular DNA. In the presence of SPION^LA1^ and SPION^LA2^ large structures developed, which contained iron oxide nanoparticle aggregates arranged in large rows (dark structures in bright field). Around the nanoparticle rows several bound viable PMN were detected by nuclear Sytox fluorescence and brightfield imaging. The aggregated nanoparticle structures were stained positive for NE, citH3, and extracellular DNA, although the green signal of the latter was partially quenched by the brownish particles. We conclude that magnet-induced primary nanoparticle agglomerates activate NET formation and build larger secondary structures glued together by aggNETs (Figures 4C,D).

### *In vivo* formation of SPION-aggregated NET-co-aggregates

**Figure 5:**
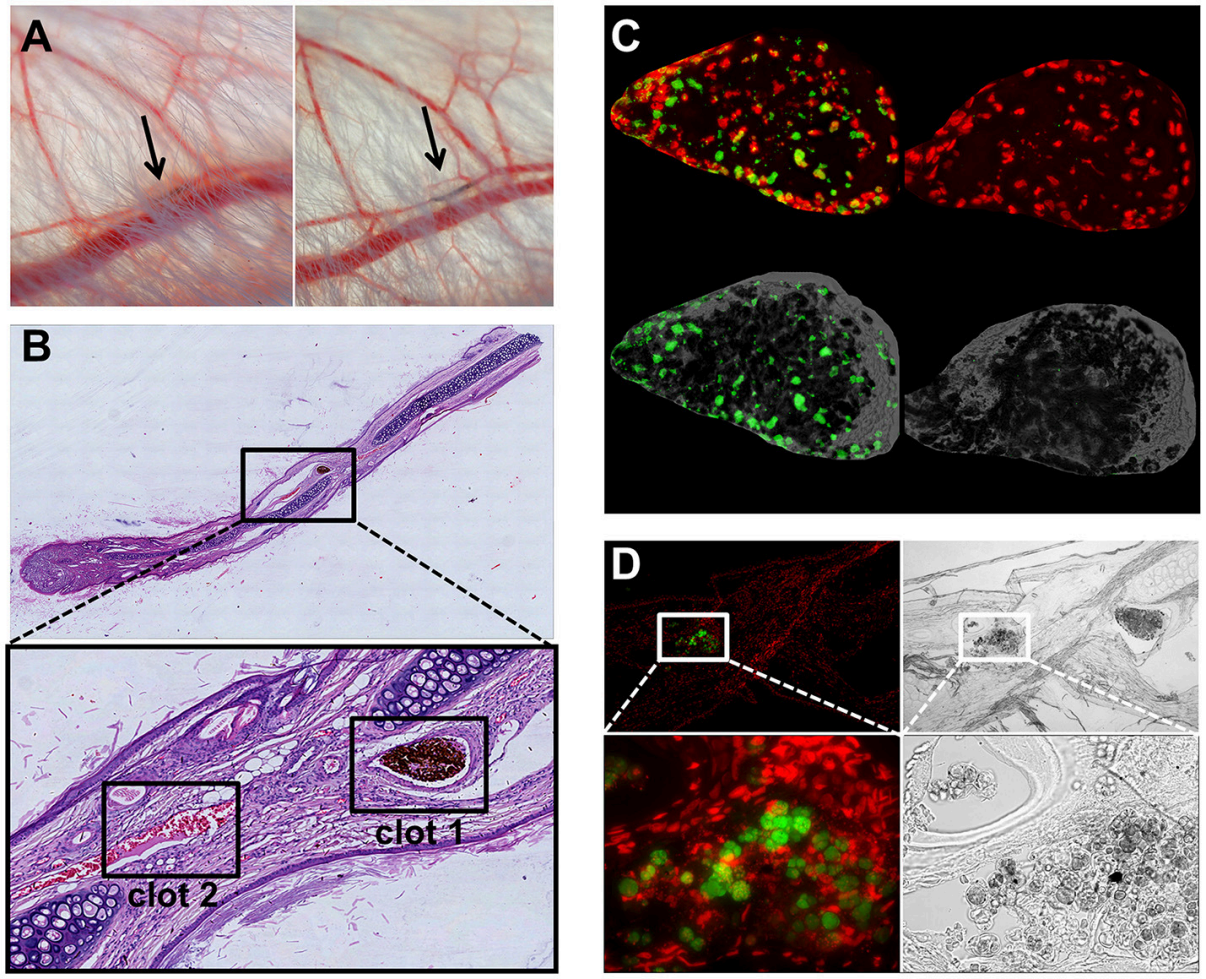
Magnetic fields induce SPION^LA1^-aggNET-co-aggregates *in vivo*. Magnetic field induces aggregation of SPION^LA1^ and aggNET in the vessel of rabbit ears, as early as 20 min after injection (left) and up to 3 days after injection (right) **(A)**. The area imaged was positioned on top of permanent neodymium magnet immediately after injection. HE staining of section from rabbit ear (Clot 1, SPION^LA1^-aggNET-co-aggregates area with initiated clot formation; Clot 2, canonical clot formed) **(B)**. Clot 1 stained for externalized DNA (green) by anti-DNA IgM antibody, and DNA (red) by PI **(C)**; Clot 2 contained Myeloperoxidase (green) and DNA (red) **(D)**.

To test whether SPION-aggNET-co-aggregates also form *in vivo* we injected SPION^LA1^ nanoparticles into the central arterial vessel of rabbit ears and subjected the distal vessel to an external magnetic field. After 20 min black agglomerations inside branches of the marginal veins of the ear were visible (Figure 5A). The intravascular agglomerates remained visible for at least 3 days after injection, when the animals were sacrificed and the ear tissue subjected to analyses by histology. Serial sections through the black agglomerates and staining with HE revealed two vascular clots with one containing a dense package of SPION^LA1^ (Figure 5B; clot 1). Staining with PI (red) and anti-dDNA IgM (green) showed that the agglomeration contained extracellular DNA (Figure 5C). Clot 2 was positive for myeloperoxidase and DNA, but did not contain any SPION^LA1^ (Figure 5D). Importantly, clot 2 was not in direct proximity to the nanoparticles, thus, neutrophils might have been activated by massive cell stress due to the downstream vessel occlusion. Alternative coating of the SPIONs with HSA or dextran (SPION^LA−HSA^ and SPION^DEX^) prevented vascular occlusions even in the presence of a magnetic field (data not shown).

## Discussion

Uncoated SPIONs tend to agglomerate due to magnetic attraction, high surface energy and van der Waals forces (13). In our study, SPION^LA1^ and SPION^LA2^ formed nanoparticle agglomerates in blood *in vitro* and *in vivo*, when brought into magnetic fields (Figures 4, 5). These instable agglomerates activated NET formation and were glued together by the extracellular chromatin. NET formation stabilized the agglomerates, leading to the formation of firm SPION-aggNETs-co-aggregates that may even occlude vessels. Thus, appropriate coating of SPIONs (SPION^LA−HSA^ or SPION^DEX^) prevents the formation of occlusive aggregates, increases the safety of the SPIONs and allows their therapeutic use.

Nanoparticles injected in the blood are known to be opsonized by serum proteins including complement compounds, immunoglobulins, fibronectin, and apolipoproteins (14). This protein corona increases biocompatibility of the nanoparticles and raises their colloidal stability (15–17). We showed that in the presence of pure plasma, plasma containing cell culture medium or whole blood nanoparticle agglomeration was prevented (Figures 1D, 2), whereas in PBS agglomeration occurred in non-sufficiently coated SPIONs (Figure 1A). After injection of nanoparticles into the blood stream, opsonisation with plasma proteins results in uptake by cells of the reticuloendothelial system (18). Thus, the majority of the nanoparticles are quickly removed by Kupffer cells of the liver and spleen (19). To circumvent fast clearance of therapeutic nanoparticles from the blood stream, coating with e.g. polyethylene glycol (PEG) is used to mask particles and to increase circulation times (20). For application of SPIONs as drug transporter in magnetic drug targeting, intraarterial injection in the tumor supplying vessels can reduce early clearance (5, 21).

Besides macrophages and monocytes, also neutrophils are involved in the phagocytic removal of pathogens. The decision if neutrophils detoxify pathogens by phagocytosis or NET formation has been shown to depend on the pathogen size amongst others (22). Here, neutrophil elastase plays a crucial role. Yeast particles for example are quickly phagocytosed, with fusion of NE-containing granules to the phagosome. When neutrophils meet a pathogen that is too big to be taken up into phagosomes, NE is slowly released into the cytosol, translocates into the nucleus and promotes chromatin decondensation (4). We observed in our experiments that NET formation depends on the agglomeration of nanoparticles. Agglomeration of insufficiently coated SPIONs was caused in the absence of stabilizing proteins (Figures 1, 2) or due to active enrichment under a magnetic field (Figures 3–5). Thus, size of the nanoparticle clusters, local nanoparticle concentration or the surface structure might play a role for the subsequent formation of NETs. In line with this, it has been shown that the topology of biomaterial implants is crucial to prevent neutrophil activation as well as acute and chronic inflammation (23). Cross-linked alginate implants attract more neutrophils than alginate injected as solution (24).

Our findings indicate that the 3D architecture of nanoparticles and their agglomerates can regulate neutrophils' responses and effector mechanisms. We showed that with magnetically induced agglomeration of nanoparticles, the formation of firm aggNET-SPION-co-aggregates is induced in a selective manner (Figure 3). This also happens in the circulation *in vivo*. These firm aggregates can form non-canonical thrombi that are able to occlude vessels (Figure 5). This is comparable to the aggNET-mediated microvascular thrombosis in sepsis (25, 26). The action of the plasma proteins, aggNETs and of NET-associated proteins (NE, histones) further increases size and stability of the thrombi. Indeed, NE can cleave and inactivate tissue factor pathway inhibitor, leading to increased pro-coagulant activity, with concomitant platelet activation and accelerated thrombus formation (27–29). In addition, aggNET-borne histones in turn activate platelets (30). The mutual activation of aggNETs and the canonical coagulation pathway further promotes coagulation and may form tight vascular occlusions, often in the capillary bed.

Besides the coagulation system, interactions of NETs with the complement system have additionally become apparent. Activated complement proteins can induce NET formation, and NETs *vice versa* can serve as a platform for complement activation (31). Previously, iron oxide-based contrast agents showed their potential to trigger hypersensitivity reactions or complement activation (32, 33). For SPION^DEX^ dedicated for magnetic resonance imaging we previously proofed the absence of complement activation *in vitro* and complement-activation related pseudoallergy (CARPA) *in vivo*, probably due to tight coverage of the iron surface by complete cross-linking of the dextran shell (8, 34), indicating the importance of proper coatings for biocompatibility. For SPIONs dedicated for magnetic drug targeting (SPION^LA−HSA^) we previously showed that an artificial albumin protein corona can colloidally stabilize iron oxide nanoparticles and increase their biocompatibility (11, 35, 36).

Here we report that SPION^LA1^ and SPION^LA2^ did not agglomerate and did not induce NETosis in plasma or serum containing cell culture media in the absence of a magnetic field. However, as soon as a magnetic field was applied, a topological structure evolved which favors NET formation and thrombogenicity. Both drawbacks were clearly prevented in preparations of SPION^LA−HSA^ and SPION^DEX^ which were coated with human serum albumin or dextran.

## Author contributions

RB developed experimental setup for animal experiments and performed animal microphotography. RB, TD, and SP performed experiments with rabbits. VV and BW processed, stained, and analyzed tissues and cells in microscopy, HU synthesized and characterized SPIONs. ZL and CJ performed flow cytometry, CA, RB, MH, and CJ planned the experiments and wrote the manuscript.

### Conflict of interest statement

The authors declare that the research was conducted in the absence of any commercial or financial relationships that could be construed as a potential conflict of interest. The reviewer GS and handling Editor declared their shared affiliation.
